# Meta-analysis of human prefrontal cortex reveals activation of GFAP and decline of synaptic transmission in the aging brain

**DOI:** 10.1186/s40478-020-00907-8

**Published:** 2020-03-05

**Authors:** Wasco Wruck, James Adjaye

**Affiliations:** Institute for Stem Cell Research and Regenerative Medicine, Medical Faculty, Heinrich Heine University, Moorenstr.5, 40225 Düsseldorf, Germany

**Keywords:** Prefrontal cortex, Aging, Sex-specific, Meta-analysis, Transcriptome

## Abstract

Despite ongoing research efforts, mechanisms of brain aging are still enigmatic and need to be elucidated for a better understanding of age-associated cognitive decline. The aim of this study is to investigate aging in the prefrontal cortex region of human brain in a meta-analysis of transcriptome datasets. We analyzed 591 gene expression datasets pertaining to female and male human prefrontal cortex biopsies of distinct ages. We used hierarchical clustering and principal component analysis (PCA) to determine the influence of sex and age on global transcriptome levels. In sex-specific analysis we identified genes correlating with age and differentially expressed between groups of young, middle-aged and aged. Pathways and gene ontologies (GOs) over-represented in the resulting gene sets were calculated. Potential causal relationships between genes and between GOs were explored employing the Granger test of gene expression time series over the range of ages. The most outstanding results were the age-related decline of synaptic transmission and activated expression of glial fibrillary acidic protein (GFAP) in both sexes. We found an antagonistic relationship between calcium/calmodulin dependent protein kinase IV (*CAMK4)* and *GFAP* which may include regulatory mechanisms involving cAMP responsive element binding protein (CREB) and mitogen-activated protein kinase (MAPK, alias ERK). Common to both sexes was a decline in synaptic transmission, neurogenesis and an increased base-level of inflammatory and immune-related processes. Furthermore, we detected differences in dendritic spine morphogenesis, catecholamine signaling and cellular responses to external stimuli, particularly to metal (Zinc and cadmium) ions which were higher in female brains.

## Introduction

Mechanisms associated with time-dependent physical decline, i.e., *aging* are complex and despite its omnipresence in biological organisms our understanding of it is still not complete. Recently, López-Otín et al. proposed nine hallmarks of aging [35] into: (i) the four causative hallmarks *Genomic instability*, *Telomere attrition*, *Epigenetic alterations* and *Loss of proteostasis*, (ii) the three hallmarks as response to damage *De-regulated nutrient sensing*, *Mitochondrial dysfunction* and *Cellular senescence* and (iii) the two integrative hallmarks *Stem cell exhaustion* and *Altered intercellular communication* which as a result from the others are responsible for functional decline. Roles of oxidative stress in aging have been manifested in a large body of publications, e.g. [7] but have also been challenged recently [26]. Hekimi et al. do not consider reactive oxygen species (ROS) as the primary cause of aging but rather as a mediator of stress response to age-dependent damage. Brink et al. propose the metabolic stability theory of aging, which postulates that the aging process depends on maintaining stable concentrations of reactive oxygen species (ROS) and other critical metabolites [7].

The rate of aging varies in an organ-specific manner ([7]). The observation that adult brains do not grow further led to the notion that neurogenesis declines with age, this however remains contentious. The dogma of no postnatal neurogenesis was rejected as far back as the 1990s by studies dating back to the 1960s [2] also finding neurogenesis in adult brains [32]. However, the level of neurogenesis in the adult brain is at a low level and therefore the established conclusions that most of the cognitive tasks are controlled by synapsis dynamics still holds true. We previously described that aging is the most important factor in the etiology of Late-onset-Alzheimer’s disease (LOAD) and identified gene-regulatory networks in hippocampus correlating with metabolic instability and oxidative stress [53]. The distinction between disease-associated and aging-related phenotypes is important. Whilst AD and Mild cognitive impairment (MCI) are associated with the loss of neurons, age-related cognitive impairment (ARCI) is not characterized by neuronal loss but rather by changes in the dynamics of synapses. Synapse dynamics depend on three types of dendritic spines: stubby, thin and mushroom spines [25]. Mushroom spines are considered responsible for long-term memory while thin spines are considered to arrange synapses for flexible cognitive tasks [6]. Morrison et al. reported that these thin spines were found to be reduced during aging and their density showed the highest correlation to performance on a cognitive task (DNMS: delayed nonmatching-to-sample) in non-human primates [39]. Mostany et al. reported that old mice possess the same spine density but a higher stability of spines when compared to mature mice and therefore might imply that age-related deficits in sensory perception are rather associated with alterations in the size and stability of spines and boutons than with the loss of synases [40]. Dendritic spine density can be increased by estradiol [52], thus, hormonal balance plays an important role in cognitive performance. Furthermore, age-associated decrease in hormone levels can also be considered as a reason for cognitive decline in elderly persons. In females after menopause, cognitive performance has been shown to be improved by estrogen-replacement therapies [45]. The body of literature is much smaller for males but regulation of dendritic spine density by testosterone has also been reported [20].

The role of astrocytes in healthy and diseased brain is gaining more attention due to the observation that astrocytes play major roles in synaptic transmission, information processing, energy supply and control of blood flow [46]. Analogous to inflammation, the re-activation of astrocytes in response to neural injury is indispensable, and uncontrolled reactivation can be detrimental- ultimately leading to brain disease. In this study, we investigated changes in the transcriptomes, associated pathways and gene ontologies in the brains of males and females during aging by a meta-analysis of 591 datasets from prefrontal cortex biopsies taking into account sex-specific differences and commonalities.

## Materials and methods

### Data analysis

Transcriptome datasets of 591 pre-frontal cortex biopsies measured on several Affymetrix microarray platforms and via rnaSeq (Illumina HiSeq) were downloaded from NCBI GEO (Supplementary Table 1). These datasets originate from studies by Narayan et al. [41], Barnes et al. [4], Lu et al. [36], Lanz et al. [34], Chen et al. [10], Hagenauer et al. [24] and Cheng et al. [11]. Table 1 shows the distribution of the datasets between female and male samples and over age groups. All data were read into R/Bioconductor [21] and normalized together employing the R package inSilicoMerging [48] parametrized to use the Combat method in order to remove batch effects. For the generation of dendrograms, genes were filtered with a coefficient of variation greater than 0.1 and afterwards subjected to hierarchical cluster analysis using complete linkage as an agglomeration method and Pearson correlation as similarity measure. Colour bars indicative of aging or sex were added to the dendrograms via the R package dendextend [19]. Genes for Principal Component Analysis (PCA) were filtered analogously as for dendrograms and afterwards the PCA of their logarithmic (base 2) gene expression was calculated using the R function *prcomp*. Based on the PCA, gene expression was predicted employing the function *predict* and the prediction for the first two components was plotted with age- or sex -specific colour schemes. The proportions of variance of the first two principal components were determined using the attribute named *importance* from the summary function of the *prcomp* object. The screeplot was generated with the *plot*() method of the *prcomp* object. Genes with most influence on the principal components were found with the function *get_pca_var*() from the R package factoextra [30] and plotted with the package corrplot [50].

**Table 1:** Characteristics of PFC datasets, distribution of female and male samples and in age groups

Dataset	Age < 30	Age 30–65	Age > 65	Male	Female	M/F	Total
GSE21138	6	19	4	24	5	4.80	29
GSE21935	2	5	12	10	9	1.11	19
GSE53890	8	12	21	20	21	0.95	41
GSE53987	1	17	1	10	9	1.11	19
GSE71620	52	316	52	332	88	3.77	420
GSE92538	3	37	15	35	20	1.75	55
GSE106669	1	3	4	8	8	1.00	8
Total	73	409	109	439	160	2.74	591

### Detection of age-associated gene expression

For each gene-*g,* the Pearson correlation with age *r*_*gxa*_ was calculated with the R function *cor()* using the normalized logarithmic (base 2) gene expression as *x* and the age of the corresponding individual as *a*. The corresponding *p*-value was determined via the R function *cor.test()*. The values were calculated separately for male and female prefrontal cortex gene expression. Plots over age were generated from the logarithmic normalized expression data with the R functions *matplot()* and *matlines()* fitting a third order polynomial model to the gene expression data for the regression curve.

### Pathway and GO over-representation plots

Over-represented KEGG pathways were calculated employing the R built-in hypergeometric test. Pathway annotations were downloaded from the KEGG database in March 2018 [29]. Over-represented GOs were determined via the R package GOStats [16]. The *n* most significantly over-represented KEGG pathways and GOs (*n* = 20) were plotted in a special dot plot indicating p-value of the hypergeometric test, number of significant genes per pathway/GO and gene ratio (ratio of significant genes to all genes in the pathway/GO) using package ggplot2 [51].

### Protein interaction networks

Human protein interactions and interactors of interactors were extracted from the Biogrid database version 3.4.161 [9] using genes significantly correlated and anti-correlated genes (Bonferroni-corrected *p* < 0.05). The resulting complex network was reduced to the shortest paths between the original set via the method get.shortest.paths from R package igraph [12] and was plotted employing community cluster networks identifying communities with more internal than external links via function *cluster-edge-betweenness*.

### Time series analysis

In order to identify genes associated with *GFAP,* Pearson correlation coefficient of the expression of all genes to the expression of *GFAP* was calculated. The genes with the highest positive or negative correlation were filtered and subjected to time series analysis. As it was obviously not possible to generate the time series from multiple measurements at the same individual during aging they represent only a model of aging reconstructed from single measurements at multiple individuals. Thus, the measurements include gene expression variability between individuals. In order to smoothen the time series, a polynomial of degree three was fitted to the data. For follow-up analyses a stationary time series was needed. We used the function *ndiffs()* from the R package *forecast* [28] to check the stationarity of the time series and that no further differentiation was needed. The function was parametrized to use the Kwiatkowski-Phillips-Schmidt-Shin (KPSS) test with the null hypothesis of a stationary root. We adapted the Granger test which tests causality between time series [22] to test Granger causality between these time series reconstructed from gene expression measured in post-mortem brain biopsies from individuals comprising a full spectrum of ages at death. We test the null hypothesis that the time series *g* of one gene does not cause the time series *h* of another gene. This is tested via an auto-regression model of *h* to which lagged values of *g* are added so that the null hypothesis is equivalent to test the coefficients b_i_ for equality to zero:

1 $$ {h}_t=\sum \limits_{i=1}^L{a}_i{h}_{t-i}+\sum \limits_{i=1}^L{b}_i{g}_{t-i}+{\upvarepsilon}_t $$

2 $$ {H}_0:{b}_1=\cdots ={b}_L=0\ \left(\mathrm{gene}\ h\ \mathrm{does}\ \mathrm{not}\ \mathrm{Granger}\ \mathrm{cause}\ \mathrm{gene}\ g\right) $$

Here, a_i_ are coefficients of the auto-regression model of *h* and b_i_ coefficients for the added lagged values of *g*, ε_t_ is the error. The time series of the expression of these genes during aging compared to the *GFAP* time series were tested for Granger causality with the function grangertest from the R package lmtest [56].

### Time series analysis on the GO level

The above described time series analysis was extended in order to uncover relationships between GOs and between genes and GOs. To achieve this, the means of the expression values of genes significantly correlated or anti-correlated with age and associated with a GO were calculated. The time series consisting of these mean values was considered a consensus time series for the dedicated GO. Let *A* be the set of ages for which data exists and *G*_*gu*_ and *G*_*gd*_ be the sets of genes significantly correlated and anti-correlated with age from the GO *g*:

3 $$ {G}_{gu}=\left\{ genes\ correlated\ (up)\  with\  age\  in\  GO\ g\right\} $$

4 $$ {G}_{gd}=\left\{ genes\ anticorrelated\ (down)\  with\  age\  in\  GO\ g\right\} $$

5 $$ {X}_u=\left\{{X}_{aui};a\in A;i\in {G}_{gu}\right\} $$

6 $$ {X}_d=\left\{{X}_{adi};a\in A;i\in {G}_{gd}\right\} $$

The consensus time series ${\overline{X}}_{gu}$ and ${\overline{X}}_{gd}$ for GO *g* are then:

7 $$ {\overline{X}}_{gu}=\frac{1}{\left|{G}_{gu}\right|}\sum \limits_{i\in {G}_{gu}}{X}_{aui} $$

8 $$ {\overline{X}}_{gd}=\frac{1}{\left|{G}_{gd}\right|}\sum \limits_{i\in {G}_{gd}}{X}_{adi} $$

Granger causality between this GO consensus time series and other significantly over-represented GO consensus time series was determined. Furthermore, Granger causality tests between genes of interest, e.g. *GFAP,* and GO consensus time series were carried out.

## Results

### Sex differences are more prominent than age differences in prefrontal cortex

**Fig. 1:**
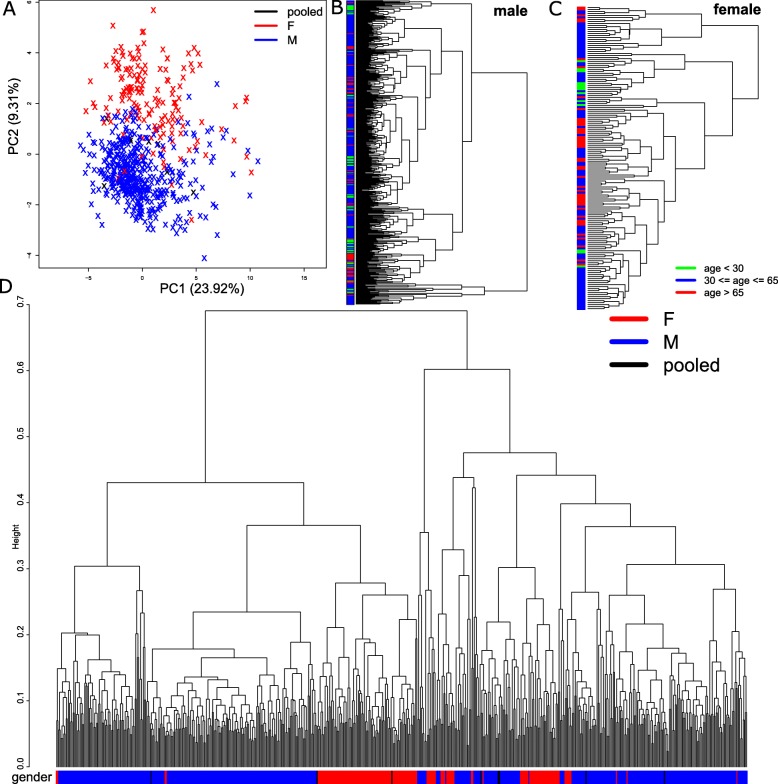
Sex differences are more prominent than age differences in prefrontal cortex. **a** Principal component analysis (PCA) of pre-frontal cortex gene expression data shows a separation between female (red) and male (blue). Pooled samples of both sexes are located in the middle between male and female. Sex-specific clustering gives heterogeneous images of age groups where only tendencies for clusters of younger samples to the left and more older samples to the right can be identified in male (**b**) as well as female (**c**). **d** The dendrogram of male and female samples together shows large sex-specific contiguous regions

Five hundred ninety-one prefrontal cortex (PFC) biopsies-derived transcriptome datasets (Supplementary Table 1) from control donors without diagnosed disease were downloaded from National Center for Biotechnology information (NCBI) Gene Expression Omnibus (GEO). After normalization and batch effect adjustment, the datasets were characterized via Principal component analysis (PCA). The plot of the first two components explaining the highest percentage of variance (Fig. 1a) shows a separation between female (red) and male (blue). Pooled samples containing both sexes are located in the middle between male and female. The dendrogram of male and female transcriptomes essentially confirms the sex effect showing large sex-specific contiguous regions (Fig. 1d). Trying to find reasons for this sex effect, we directly compared male and female transcriptomes and found that the most significantly differentially expressed genes were located on the sex chromosomes (Supplementary Table 2). Based on this, we performed the follow-up analyses in a sex-specific manner. Separate cluster analyses for male and female showed predominantly age-independent clusters with some sub-clusters possessing tendencies for younger or older samples in male (Fig. 1b) as well as female (Fig. 1c).

### Sex-specific differential expression between young, middle-aged and old

**Fig. 2:**
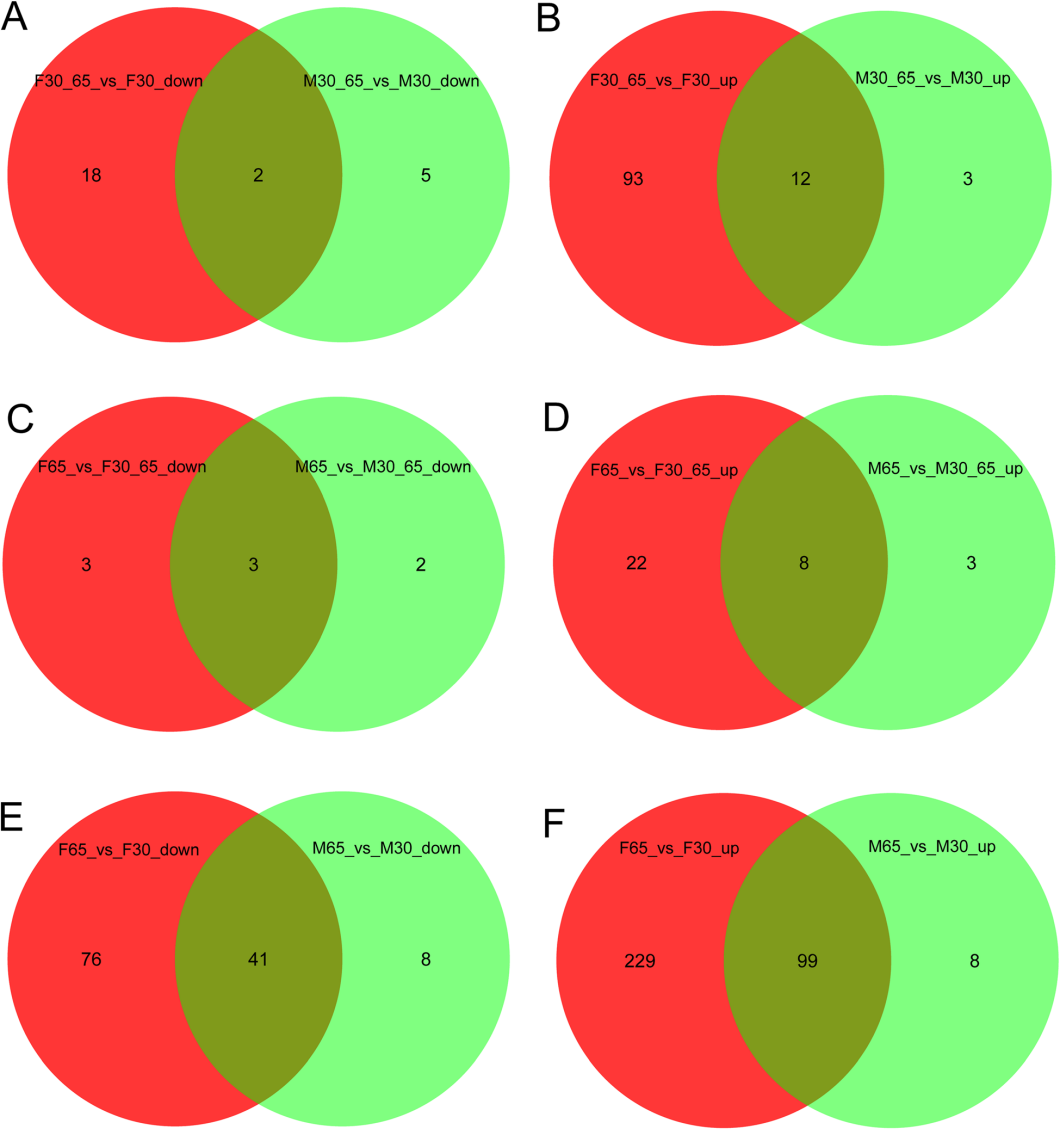
Most genes were differentially expressed between groups of age > 65 and age < 30. Down-regulated (**a**, **c**, **e**) and up-regulated (**b**, **d**, **f**) genes were compared in venn diagrams between female (red circles) and male (green circles) prefrontal cortex. Age was grouped in a sex-specific way into age < 30 (F30, M30), 30 < age < 65 (F30_65, M30_65) and age > 65 (F65, M65). **a** Genes down-regulated in F30_65 vs. F30 were compared with genes down-regulated in M30_65 vs. M30. **b** Genes up-regulated in F30_65 vs. F30 were compared with genes up-regulated in M30_65 vs. M30. **c** Genes down-regulated in F65 vs. F30_65 were compared with genes down-regulated in M65 vs. M30_65. **d** Genes up-regulated in F65 vs. F30_65 were compared with genes up-regulated in M65 vs. M30_65. **e** Genes down-regulated in F65 vs. F30 were compared with genes down-regulated in M65 vs. M30. **f** Genes up-regulated in F65 vs. F30 were compared with genes up-regulated in M65 vs. M30. Most genes were differentially expressed between the more distant groups of age > 65 and age < 30 while in the comparisons between the adjacent age groups there were fewer genes differentially expressed. This demonstrates continuous long-term changes in gene expression. From the fewer genes differentially expressed in male biopsies most were in common with the female genes

Differentially down-regulated (ratio < 0.833, *p* < 0.05; Fig. 2a, c, e) and up-regulated (ratio > 1.2, *p* < 0.05; Fig. 2b, d, f) genes were calculated between three age groups and compared in venn diagrams between female (red circles) and male (green circles) prefrontal cortex. Sex-specific age groups contained age younger than 30 (F30, M30), age between 30 and 65 (F30_65, M30_65) and age over 65 (F65, M65). Most genes were differentially expressed between the more distant groups of age > 65 and age < 30 while in the comparisons with the middle-aged group there were fewer genes differentially expressed. This demonstrates continuous long-term changes in gene expression. In general, in the male samples fewer genes were differentially expressed than in females which may partly be due to the different sample numbers in male and female. Thus, except for the comparison of down-regulated middle-aged vs. young (Fig. 2a) more genes found in male biopsies overlapped with female genes than were exclusive in male. This overlap between male and female shows congruency between the sexes thus seeming to contradict the sex-effect found previously in the PCA plot and dendrogram (Fig. 1a, d). An explanation could be that while most genes are expressed similarly in male and female, sex-specific expression is mostly induced by genes on the sex chromosomes. As more detailed functional annotation of genes become available later in this study, analysis carried out so far revealed that *GFAP* is up-regulated with increasing age whereas *ALB1* and *CX3CR1* are down-regulated with age in both sexes. For the complete gene lists corresponding to the venn diagram analyses refer to Supplementary Table 3.

### Genes down-regulated during aging are associated with synaptic processes

**Fig. 3:**
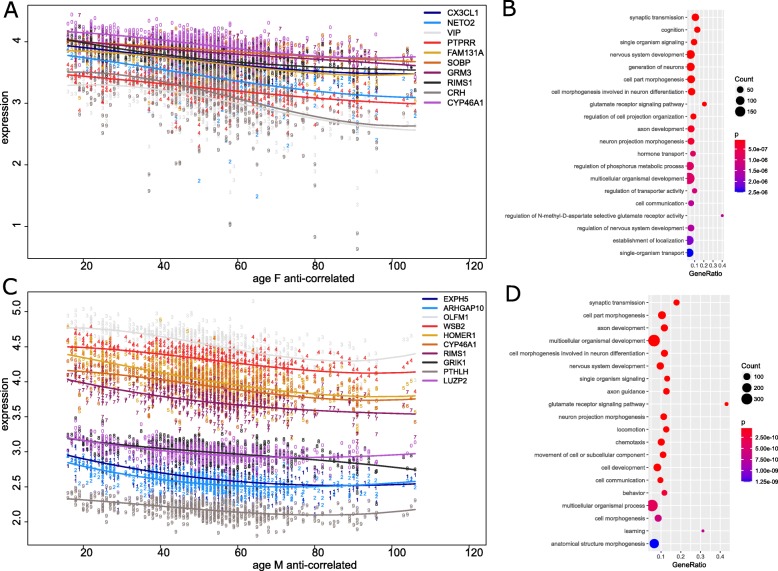
Genes down-regulated during aging are associated with synaptic processes . Gene ontologies (GOs) of genes which were most significantly anti-correlated with age were analyzed separately for male and female prefrontal cortex. GO terms related to synaptic signaling were found in both sexes

For each gene the Pearson correlation coefficient and corresponding *p*-value of its expression with the age of the associated individuals was calculated separately for male and female prefrontal cortex (Supplementary Table 4). Figure 3a shows a plot of the expression of the ten genes most significantly anti-correlated with age in female ranked by the correlation, Fig. 3c analogously in male. *CYP46A1* (F: *r* = − 0.57, M: *r* = − 0.53) and *RIMS1* (F: *r* = − 0.58, M: *r* = − 0.51) were among these in both sexes, *CX3CL1* (*r* = − 0.61) was lowest in female, *EXPH5* (*r* = − 0.58) was lowest in male (Supplementary Table 4). Gene ontologies (GOs) of genes which were most significantly anti-correlated with age (Bonferroni-corrected *p* < 0.05, *r* < − 0.1) were analyzed separately for male and female prefrontal cortex. The 20 most significantly over-represented GO terms (GO type Biological process) are shown in dot plots indicating p-value of hypergeometric test, gene count and ratios of genes annotated with the GO term (Fig. 3b for female, Fig. 3d for male). In both sexes, GO terms related to synaptic signaling were found as most significant (F: *p* = 1.2E-19, M: *p* = 8.1E-21, Supplementary Table 5, Fig. 3b, d). Numerous neuron-related GO terms were detected as down-regulated with age – amongst these are, *axon development, nervous system development, generation of neurons, glutamate receptor signaling pathway, cell morphogenesis involved in neuron differentiation*. Additionally, further functional groups including hormones, glucocorticoids, catecholamine, neurogenesis and synapse related processes such as Long-Term-Potentiation (LTP), cAMP signaling, dendritic spines, could be identified among the significant GO terms (Table 2). While most of these GO terms provide further detail for the central finding of age-related reduction of synaptic transmission hormones and dendritic spines may be causative. The expression levels of numerous hormones such as estradiol decrease with age and are known to influence synaptic plasticity by changing the numbers and characteristics of dendritic spines.

**Table 2:** Selected groups of significant GO terms overrepresented in genes anti-correlated with age in female and male

Group	Term_female	*P* value_F	Term_male	*P* value_M
Catecholamine	catecholamine uptake involved in synaptic transmission	1.47E-04	catecholamine secretion	5.33E-04
	cellular response to catecholamine stimulus	8.13E-04		
	catecholamine transport	1.47E-03		
	regulation of catecholamine secretion	1.76E-02		
	catecholamine binding	4.99E-02		
Hormone	hormone transport	8.20E-07	hormone transport	6.75E-07
	regulation of hormone secretion	2.56E-06	regulation of hormone secretion	3.64E-06
	peptide hormone secretion	4.88E-05	peptide hormone secretion	1.77E-05
	response to peptide hormone	1.01E-03	response to peptide hormone	2.62E-05
	hormone-mediated apoptotic signaling pathway	4.18E-03	cellular response to hormone stimulus	1.11E-04
	cellular response to hormone stimulus	5.97E-03	positive regulation of peptide hormone secretion	9.68E-03
	negative regulation of peptide hormone secretion	6.89E-03	hormone-mediated apoptotic signaling pathway	1.06E-02
	regulation of intracellular steroid hormone receptor signaling pathway	1.97E-02	thyroid hormone transport	1.72E-02
	neuropeptide hormone activity	2.45E-06	positive regulation of corticosteroid hormone secretion	3.41E-02
			regulation of intracellular steroid hormone receptor signaling pathway	3.90E-02
			cellular response to parathyroid hormone stimulus	4.42E-02
			neuropeptide hormone activity	6.12E-05
			peptide hormone receptor binding	7.63E-03
Corticoid	positive regulation of glucocorticoid receptor signaling pathway	1.94E-05	positive regulation of glucocorticoid receptor signaling pathway	8.12E-05
	corticosteroid receptor signaling pathway	1.17E-03	corticosteroid receptor signaling pathway	6.59E-03
			positive regulation of corticosteroid hormone secretion	3.41E-02
Neurogenesis	positive regulation of neurogenesis	2.84E-05	positive regulation of neurogenesis	1.51E-05
	negative regulation of neurogenesis	3.31E-03		
cAMP	regulation of cAMP biosynthetic process	7.42E-05	regulation of cAMP biosynthetic process	6.39E-07
	negative regulation of cAMP metabolic process	7.43E-04	negative regulation of cAMP metabolic process	5.18E-05
	positive regulation of cAMP metabolic process	1.01E-02	positive regulation of cAMP metabolic process	4.47E-04
			hippocampus development	7.40E-04
			cAMP-mediated signaling	9.50E-04
			negative regulation of cAMP-mediated signaling	3.86E-03
			cAMP catabolic process	4.08E-02
LTP	positive regulation of long-term synaptic potentiation	6.25E-04	long-term synaptic potentiation	5.54E-06
	long-term synaptic potentiation	4.07E-03	positive regulation of long-term synaptic potentiation	2.49E-03
Dendritic spine	negative regulation of dendritic spine development	2.02E-03	dendritic spine morphogenesis	4.75E-05
	dendritic spine organization	3.74E-03	regulation of dendritic spine morphogenesis	2.44E-03
	regulation of dendritic spine morphogenesis	5.59E-03	negative regulation of dendritic spine development	7.75E-03
	dendritic spine development	9.70E-03	positive regulation of dendritic spine morphogenesis	1.03E-02
	positive regulation of dendritic spine morphogenesis	3.38E-02	dendritic spine	4.60E-09
	dendritic spine	5.69E-05	dendritic spine head	2.26E-03
	dendritic spine head	5.73E-04		
	dendritic spine membrane	2.17E-02		

### Genes up-regulated during aging are associated with the astrocyte marker GFAP and inflammation

**Fig. 4:**
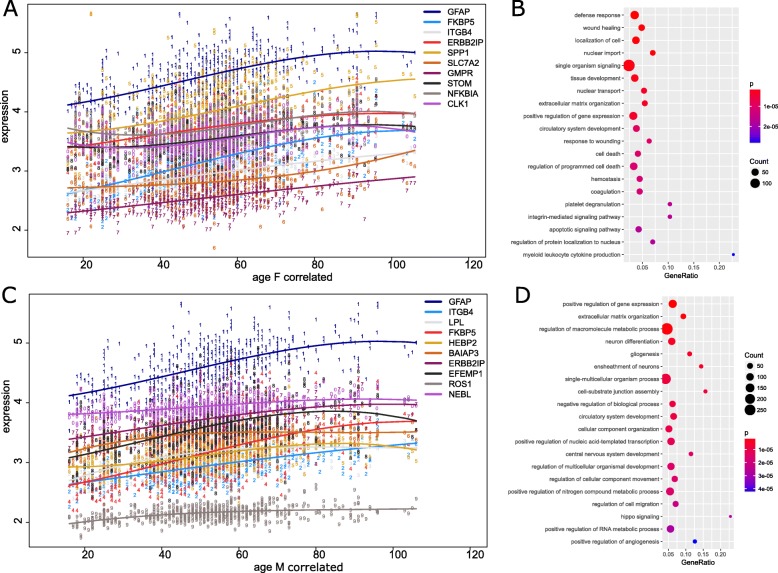
Genes up-regulated during aging are associated with the astrocyte marker GFAP and inflammation. Gene ontologies (GOs) of genes which were most significantly correlated with age were analyzed separately for male and female pre-frontal cortex. In both sexes the astrocyte marker GFAP has the highest correlation and GO terms related to inflammation were predominant

Based on the Pearson correlations with age (Supplementary Table 4) the ten genes most significantly correlated with age were plotted in female (Fig. 4a) and male (Fig. 4c). *GFAP* (F: *r* = 0.62, M: *r* = 0.55), FKBP5 (F: *r* = 0.62, M: *r* = 0.47), ITGB4 (F: *r* = 0.56, M: *r* = 0.51) and *ERBB2IP* (F: *r* = 0.56, M: *r* = 0.44) were among these in both sexes, *GFAP* was highest in both female and male (Supplementary Table 4). GOs of genes which were most significantly correlated with age (Bonferroni-corrected *p* < 0.05, *r* > 0.1) were analyzed separately for male and female prefrontal cortex. The dot plots in Fig. 4b (female) and Fig. 4d (male) show the 20 most significantly over-represented GO terms (as in Fig. 3b, d). The GO terms *extracellular matrix organization* and *circulatory system development* and *positive regulation of gene expression* (probably due to selection of upregulated genes) appear in both sexes while the rest of these top 20 terms differ between sexes. Further functional GO term groups shown in Table 3 include *immunity*, *inflammation*, *ROS* and *integrin-associated terms*. The immunity and inflammation-related terms are much more abundant in females, thus implying probable sex-associated regulation of inflammation and immune response during aging.

**Table 3:** Selected groups of significant GO terms overrepresented in genes correlated with age in female and male

Group	Term_female	*P* value_F	Term_male	*P* value_M
Immunity	immune response	2.16E-04	negative regulation of immune system process	1.25E-02
	regulation of immune system process	2.58E-04		
	regulation of production of molecular mediator of immune response	7.18E-03		
	positive regulation of cytokine production involved in immune response	1.67E-02		
	leukocyte mediated immunity	2.98E-02		
	immune system process	3.53E-02		
	activation of immune response	3.67E-02		
	regulation of innate immune response	4.00E-02		
	immunoglobulin secretion	4.47E-02		
	negative regulation of immune response	4.80E-02		
Inflammation	positive regulation of inflammatory response	6.21E-03	acute inflammatory response	1.69E-02
	regulation of inflammatory response	6.90E-03		
	acute inflammatory response	1.12E-02		
ROS	regulation of reactive oxygen species biosynthetic process	5.79E-04	positive regulation of reactive oxygen species metabolic process	2.63E-03
	positive regulation of reactive oxygen species metabolic process	2.72E-03	regulation of reactive oxygen species biosynthetic process	6.00E-03
	response to oxidative stress	2.22E-02	response to oxidative stress	3.94E-02
	intrinsic apoptotic signaling pathway in response to oxidative stress	3.64E-02		
Integrin-associated terms	integrin-mediated signaling pathway	1.67E-05	integrin-mediated signaling pathway	2.72E-04
	integrin binding	7.38E-03	cell adhesion mediated by integrin	3.53E-02
			integrin binding	2.83E-04

### Aging-related changes in pathways

**Fig. 5:**
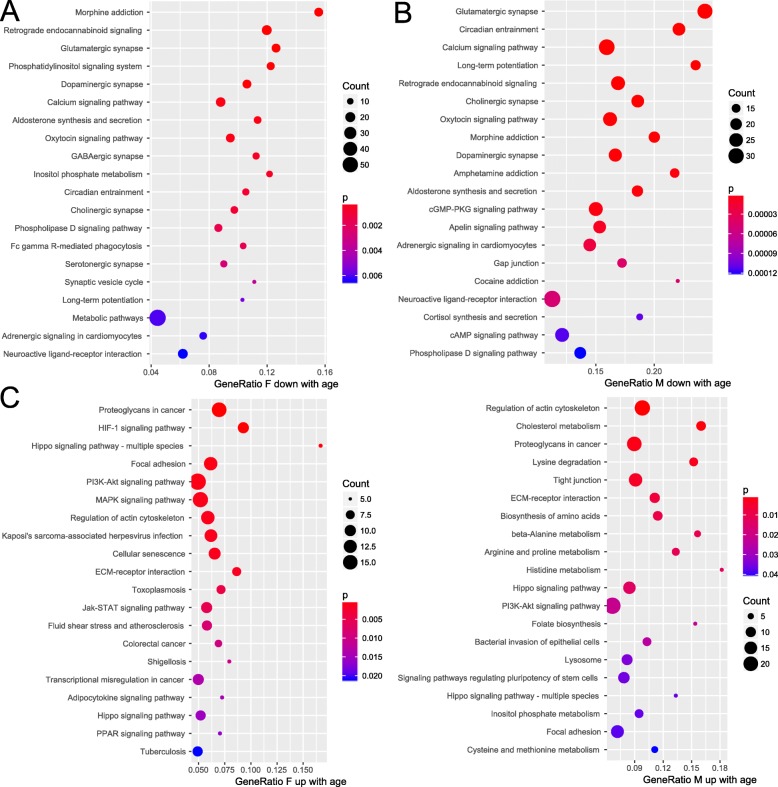
Pathways down-regulated during aging are associated with various types of synapses, calcium signaling and long-term potentiation while up-regulated pathways are associated with the extracellular matrix, cytoskeleton, Hippo- and PI3K-Akt signaling. KEGG pathways of genes which were most significantly correlated and anti-correlated with age were analyzed separately for male and female prefrontal cortex. In both sexes pathways related to various types of synapses, calcium signaling and long-term potentiation were found overrepresented in the genes anti-correlated with age. In the genes correlated with age pathways associated with the extracellular matrix, cytoskeleton, Hippo- and PI3K-Akt -signaling are overrepresented

Sex-specific pathway analysis of genes which were most significantly correlated (Bonferroni-corrected *p* < 0.05, *r* > 0.1) and anti-correlated (Bonferroni-corrected *p* < 0.05, *r* < − 0.1) revealed several over-represented KEGG pathways [29]. The dot plots in Fig. 5 show the 20 most significantly over-represented KEGG pathways for each of these four analyses. The full pathway analysis results are provided in Supplementary Table 6. Down-regulation (anti-correlation) with age was associated with various types of synapses, calcium signaling and long-term-potentiation in both sexes (Fig. 5a, b). To elucidate further causes leading to decline of synaptic transmission pathways *Cortisol synthesis and secretion* (F:*p* = 0.02,q = 0.1; M:*p* = 0.0001,q = 0.001), *cAMP signaling* (F:*p* = 0.05, q = 0.23; M:*p* = 0.0001,q = 0.001) and *Estrogen signaling* (F:*p* = 0.03,q = 0.17;M:*p* = 0.005,q = 0.03) were found (Fig. 5b, Supplementary Table 6A,B). Estrogens have been reported to regulate dendritic spine density [52].

Amonsgt the genes correlated with age, we identified over-represented pathways associated with the extracellular matrix, cytoskeleton and Hippo- and PI3K-Akt –signaling (Fig. 5c, d). For the detailed pathways see (Fig. 5c, d, Supplementary Table 6C, D): *Regulation of actin cytoskeleton* (F:*p* = 0.001,q = 0.02; M:*p* = 0.0004,q = 0.09), *Proteoglycans in cancer* (F:*p* = 7.6E-05,q = 0.01; M:*p* = 0.002,q = 0.16), *ECM-receptor interaction* (F:*p* = 0.001,q = 0.02; M:*p* = 0.007,q = 0.26), Hippo signaling (F:*p* = 0.0004,q = 0.02; M:*p* = 0.01,q = 0.27), and *PI3K-Akt signaling* (F:*p* = 0.0009,q = 0.02; M:*p* = 0.02,q = 0.38). Interestingly, the *cholesterol metabolism* pathway was over-represented in male (*p* = 0.001, q = 0.12) but not in female (*p* = 0.09, q = 0.28).

### Protein interaction networks

**Fig. 6:**
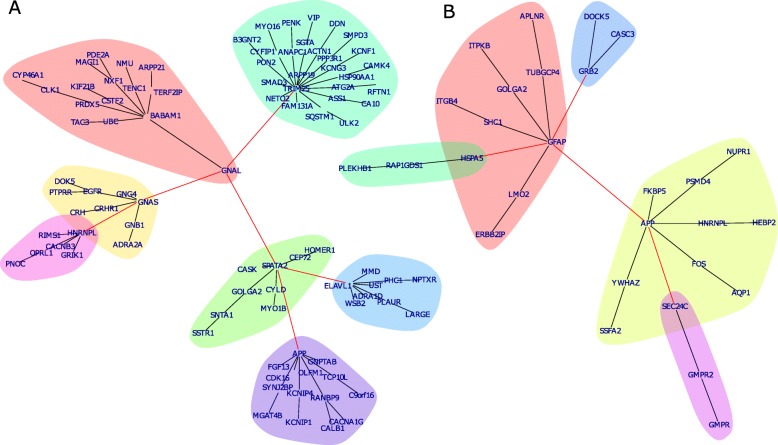
Protein interaction networks highlight major role of astrocyte marker GFAP during aging. **a** Protein interaction network of proteins coded by genes down-regulated with age based on interactions from the BioGrid database. G-protein subunit alpha L (GNAL) is at the center of several clusters which are characterized by hub proteins BABAM1 (red), GNAS (yellow), TRIM25 (petrol), SPATA2 (green), APP (violet) and ELAVL1 (blue). **b** Protein interaction network of proteins coded by genes up-regulated with age based on interactions from the BioGrid database. The astrocyte marker-GFAP, has a central role and is directly connected to APP

Protein interaction networks were generated based on interactions from the BioGrid database (version 3.4.161) using proteins coded by genes going down with age as filtered with the criteria of a Pearson correlation < − 0.4 and a Bonferroni adjusted *p* < 0.05 (Fig. 6a). G protein subunit alpha L (GNAL; *r* = − 0.4, *p* = 4E-18 in male; *r* = − 0.46, *p* = 2E-09 in female; Supplementary Table 4A) is at the center of this network accounting for the involvement of G-protein and its receptors in most physiological responses to hormones, neurotransmitters. Several clusters are arranged around GNAL which are characterized by hub proteins BABAM1 (red), GNAS (yellow), TRIM25 (petrol), SPATA2 (green), APP (violet) and ELAVL1 (blue). Analogously to the downregulated genes, the protein network of the upregulated genes was generated by filtering with the same *p*-value but with a Pearson correlation > 0.4 (Fig. 6b). The reactive astrocyte marker GFAP – coded by the gene with the highest correlation with age (*r* = 0.55 in male, *r* = 0.62 in female; Supplementary Table 4A) - has a central role in this network and is directly connected with APP.

### Time series analysis of GFAP

**Fig. 7:**
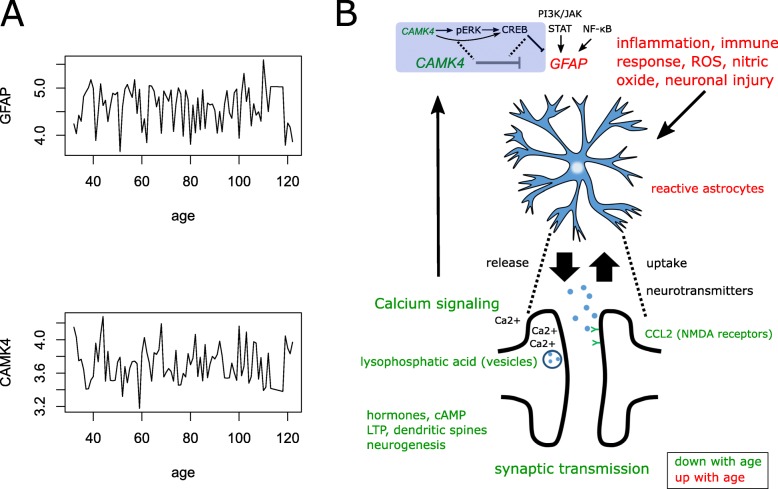
Astrocyte marker GFAP has the highest correlation with prefrontal cortex aging and depends causally on CAMK4 in the time series. **a** The plots display time series of the genes GFAP possessing the highest positive and CAMK4 possessing negative correlation with age. The Wald test shows that the time series of CAMK4 is causative for GFAP time series. **b** A simplified scheme illustrates activation of astrocytes (marker GFAP) by inflammation, ROS and neuronal injury regulating uptake and release of neurotransmitters responsible for synaptic transmission. GFAP is regulated by CAMK4 – possibly via pERK and CREB (blue shading) - which is going down during aging and is downstream of Calcium signaling pathway. Down-regulation during aging is marked with green colour, up-regulation with red colour

Time series of *GFAP* gene expression with age were analyzed and compared with highly age-correlated and anti-correlated candidate genes with the aim of finding possible causal relationships. The gene *CAMK4* was found causative for the *GFAP* time series with the Granger causality test from the R package lmtest (*p* = 0.015). The test for causality in the opposite direction was not significant (*p* = 0.52) indicating that regulation by a third gene can be excluded. The time series of *GFAP* possessing the highest positive and *CAMK4* possessing negative correlation with age are plotted in Fig. 7a.

A simplified scheme (Fig. 7b) illustrates these findings together with results from the previous analyses: astrocytes (marker *GFAP*) react to neuronal injury and ROS thereby regulating inflammatory processes. They regulate the uptake and release of neurotransmitters responsible for synaptic transmission - as described by Sofroniew et al. [46]. Age-related decline of Calcium signaling decreases the levels of downstream *CAMK4* – as mentioned above Granger-causing - up-regulation of *GFAP*. CAMK4 has been reported as a direct activator of CREB via phosphorylation of the Ser-133 residue [5] or also indirectly via MAPK [54] . By analyzing the *GFAP* promoter region we identified binding sites for CREB - beside STAT and NF-κB (Supplementary Table 7) which are usually considered as regulators of GFAP expression [38]. Antagonistic regulation of CREB and GFAP has been reported [43]. The levels of hormones such as estrogen, which decline with age play a major role in regulating the density of dendritic spines and as a consequence, modulation of synaptic transmission.

### Time series analysis of GO *synaptic transmission*

In order to elucidate which processes induce synaptic transmission, we set out to test Granger causality between significant GOs and the GO *synaptic transmission*. A consensus time series for the GO *synaptic transmission* was generated by taking the mean of all time series of genes significantly up-regulated with age in this GO (for details see Methods section). Among the over-represented GO terms we looked for causal relationships to this consensus time series of *synaptic transmission* via the Granger test. Tables 4 and 5 show the up- and down-regulated GOs found causative for *synaptic transmission* this way. Interestingly, on top of the up-regulated terms in Table 4, numerous terms related to nitric oxide appear as most significant. Nitric oxide plays important roles in the nervous system and in mitochondria and has been described to mediate mitochondrial fragmentation leading to age-related neurodegenerative diseases [31]. There was also evidence that nitric oxide elevates intracellular calcium levels and thus mediates reactive astrogliosis [47]. Furthermore, in Table 4, the term *negative regulation of monocyte chemotactic protein-1 (MCP1/CCL2) production* indicates an aging-related loss of CCL2. CCL2 has been reported to be protective against neurotoxic effects of excessive glutamate at NMDA receptors [15]. El Khoury et al. additionally described protective effects of CCL2 in Alzheimer-like disease by triggering the recruitment of astrocytes and microglia and subsequent removal of Amyloid-β [14].

**Table 4:** GOs going up with age “granger-causing” GO *synaptic transmission*

Term	ts2_c_ts1_p	ts1_c_ts2_p
Nitric oxide metabolic process	**0.0027**	0.1969
Regulation of nitric-oxide synthase biosynthetic process	**0.0034**	0.1853
Positive regulation of nitric oxide biosynthetic process	**0.0081**	0.3591
Positive regulation of myelination	**0.0107**	0.0538
Negative regulation of monocyte chemotactic protein-1 production	**0.0142**	0.5627
Regulation of cell-matrix adhesion	**0.0151**	**0.0445**
Schwann cell development	**0.0207**	0.0897
Histamine secretion	**0.0370**	0.0772
Azole transport	**0.0370**	0.0772
Positive regulation of reactive oxygen species metabolic process	**0.0372**	0.3284
Macrophage activation	**0.0386**	0.9125
Skin development	**0.0436**	0.4341
Renal absorption	**0.0451**	0.9061
Response to muscle stretch	**0.0497**	0.3531

ts2_c_ts1_p: *p*-value from Granger test between time series 2 (ts2,synaptic transmission) and ts1 (order of lags = 4)

ts1_c_ts2_p: *p*-value from Granger test between ts1 and ts2 (order of lags = 4)

Significant *p*-values < 0.05 are marked in bold

**Table 5:** GOs going down with age “granger-causing” GO *synaptic transmission*

Term	ts2_c_ts1_p	ts1_c_ts2_p
Microtubule nucleation	**0.0041**	**0.0296**
Nuclear lamina	**0.0057**	0.0501
Physiological muscle hypertrophy	**0.0080**	0.1201
Cell growth involved in cardiac muscle cell development	**0.0080**	0.1201
Lysophosphatidic acid binding	**0.0092**	0.3682
Positive regulation of dendrite morphogenesis	**0.0094**	0.3850
Cyclic purine nucleotide metabolic process	**0.0098**	**0.0463**
Regulation of synaptic transmission, glutamatergic	**0.0114**	**0.0279**
Dermatan sulfate biosynthetic process	**0.0124**	0.8968
Positive regulation of cAMP metabolic process	**0.0125**	0.2016
Positive regulation of cyclic nucleotide biosynthetic process	**0.0125**	0.2016
Uropod	**0.0130**	0.7732
1-phosphatidylinositol-4-phosphate 5-kinase activity	**0.0130**	0.7732
Proton-transporting V-type ATPase, V0 domain	**0.0153**	0.0617
Regulation of cAMP biosynthetic process	**0.0160**	0.0609
Regulation of cyclic nucleotide metabolic process	**0.0160**	0.0609
Synaptic vesicle docking	**0.0180**	0.2162
Cell-matrix adhesion	**0.0183**	0.0998
rRNA 3′-end processing	**0.0186**	0.0670
Asymmetric stem cell division	**0.0192**	0.1360
Rac GTPase binding	**0.0197**	0.5859
Macromolecular complex assembly	**0.0203**	**0.0231**
Golgi cis cisterna	**0.0213**	0.1023
Endomembrane system	**0.0217**	**0.0247**
Intrinsic apoptotic signaling pathway in response to oxidative stress	**0.0222**	0.1157
Positive regulation of purine nucleotide biosynthetic process	**0.0241**	0.1792
Positive regulation of nucleotide metabolic process	**0.0249**	0.1746
Muscle tissue development	**0.0249**	0.0884
Transporter activity	**0.0250**	**0.0323**
Spindle microtubule	**0.0256**	0.0620
Striated muscle cell development	**0.0264**	0.0765
Neuromuscular junction development	**0.0269**	0.3610
Regulation of nucleotide biosynthetic process	**0.0275**	0.0665
Endoplasmic reticulum	**0.0356**	0.0755
Calcium:cation antiporter activity	**0.0385**	0.3791
Ligand-gated channel activity	**0.0400**	0.0526
Lipid modification	**0.0412**	0.3182
Phosphatidylinositol phosphorylation	**0.0429**	0.6188
Proteoglycan biosynthetic process	**0.0433**	0.7653
Regulation of purine nucleotide metabolic process	**0.0442**	0.0842
Positive regulation of nucleocytoplasmic transport	**0.0445**	0.1855
Chloride channel inhibitor activity	**0.0464**	0.2701
Regulation of synaptic vesicle transport	**0.0469**	0.0834
Glutamate secretion	**0.0480**	**0.0499**
Dendrite terminus	**0.0481**	0.7091

ts2_c_ts1_p: *p*-value from Granger test between time series 2 (ts2,synaptic transmission) and ts1 (order of lags = 4)

ts1_c_ts2_p: *p*-value from Granger test between ts1 and ts2 (order of lags = 4)

Significant *p*-values < 0.05 are marked in bold

In Table 5, the first term *microtubule nucleation* has a p-value below 0.05 in both directions indicating that a third factor may cause both. The term *lysophosphatic acid binding* has a low p-value of 0.0092 in the direction of “granger-causing” *synaptic transmission* and a relatively high p-value of 0.3682 in the opposite direction thus pointing to *lysophosphatic acid binding* as “granger-causing” *synaptic transmission*. Lysophosphatic acid has been reported to play a crucial role in the formation of vesicles at synapses [44]. The decline of this activity and its consequences in the exchange of neurotransmitters would be one coherent explanation for the decrease of synaptic transmission. Besides, many synapsis-related terms appear in Table 5 such as cAMP-, dendrite- and calcium-transport–related terms and also aging-related oxidative-stress-mediated apoptosis.

## Discussion

In this meta-analysis of transcriptomes derived from 591 prefrontal cortex biopsies, we found a gene set with significantly increasing and another with significantly decreasing expression during aging. The most outstanding gene within these gene sets was the reactive astrocyte marker *GFAP* which showed significantly increasing expression levels in the brains of aging males and females. The biological process most significantly down-regulated with aging was synaptic transmission - as expected due to its close relation to the aging-related symptoms of reduced cognitive performance. On the other hand, there is a complex causal chain of aging-related changes eventually leading to reduced synaptic transmission. We tried to elucidate these mechanistically taking into account known aging hallmarks such as metabolic instability, increasing inflammation levels and changes in intercellular communication and could identify several functional groups. Directly related to the decline of synaptic transmission was the observation of multiple types of synapses negatively correlated with aging in the pathway analyses - for example, glutamatergic, cholinergic, dopaminergic, GABAergic and serotonergic synapses. We found expression of the reactive astrocyte marker GFAP increasing with age. Of course, this has to be confirmed experimentally but however beyond the scope of this study. Astrocytes play an important role at synapses by taking up and releasing excessive neurotransmitters and transferring lactate as energy substrate [46]. Furthermore, they influence pruning and remodeling of synapses [46]. In our previous meta-analysis of human hippocampus derived biopsies, we also observed that *GFAP* expression strongly correlated with Alzheimer’s disease (AD) [53]. Thus, *GFAP* represents astroglia activation and gliosis not only in the AD-affected brain during neurodegeneration [27] but also in the disease-free aging brain.

We identified calcium signaling as decreasing with age in both sexes. Calcium has been implicated in brain aging in the Calcium dysregulation hypothesis of brain aging and AD [33]. Calcium has a 10,000 times higher concentration outside the cells and is shuffled inside through ligand-gated glutamate receptors, such as N-methyl-d-aspartate receptor (NMDAR) or various voltage-gated channels [33]. The expression levels of NMDARs decrease with age in our analysis (Fig. 3b, Supplementary Table 5A, B). We found that up-regulation of *GFAP* is connected to the decrease of *CAMK4* possibly involving gene-regulation by CREB. *CAMK4*, a member of the family of calcium/calmodulin-dependent kinases was also found oppositely regulated to *GFAP* in the neocortex of frontotemporal dementia-like mice with TDP-43 depletion [55]. Sticozzi et al. reported that nitric oxide can elevate intracellular calcium and via calcium together with the ERK/calmodulin signaling pathway can mediate reactive astrogliosis trigerred by cytokines in a specific time frame [47].

cAMP signaling decreases with age in our analysis (Table 2) and has been reported to be disrupted by aging while in the healthy brain it modulates the strength of the synapses [39]. cAMP also regulates Ca2^+^ release from the endoplasmic reticulum via ryanodine receptors (RYR) to eventually release it to the cytosol [33, 42].

A further interesting functional group declining with age are hormones (Table 2). Hormones are known to decrease during aging and hormones such as estrogen have a major impact on synaptic plasticity and cognitive performance [39].

Interestingly, the KEGG pathway- insulin secretion decreases with age in both sexes (Supplementary Table 6). It has not been fully clarified if there is insulin production in the brain but there is some evidence for it and at least it has been reported for several species [23]. An explanation for our observation is more likely the considerable overlap between down-regulated genes within the pathways of Insulin secretion and cAMP signaling which definitely plays a role in brain aging but also in pancreatic islets [18].. Frölich et al. found that insulin concentration and insulin receptor densities in the brain decrease with aging [17]. The role of insulin in aging has been assessed by a body of literature stating one major finding that insulin sensitivity is associated with longevity while insulin resistance is associated with higher mortality [1]. Evidence for the involvement of insulin in brain aging is provided by the correlation between type 2 diabetes and neurodegenerative dementias [3] and it culminates in the annotation of Alzheimer’s disease as “diabetes type 3” [13]. Anti-ageing effects have been attributed to cAMP signaling which is part of a negative feedback loop with insulin as it regulates insulin secretion in the pancreatic islets but on the other hand is itself regulated by insulin [49]. Our findings of down-regulated cAMP emphasize its role in aging because it plays a dual role in regulating insulin secretion and synapse strength.

Furthermore, the levels of reactive oxygen species (ROS) increase with age in both sexes as indicated by the significantly over-represented GOs *Regulation of ROS biosynthetic and metabolic processes* and *Response to oxidative stress* (Table 3). A large body of literature has described oxidative stress as a major player in the aging process, furthermore, Sofroniew et al. have associated increased levels of ROS as a trigger of astrogliosis [46].

We also identified down-regulation of neurogenesis with age in both sexes (Table 2). However, neurogenesis in human brain is only reported for hippocampus but not for cortex [37] and thus this finding may be rather due to similar gene expression patterns with the hippocampus or cell migration from the hippocampus. For the hippocampus, age-related decline in neurogenesis has been reported [37] what may partially contribute to diminished cognitive abilities.

Finally, we found increased inflammation and immune response predominantly in females (Table 3). These are well known aging-associated factors [7, 35] and related to reactive astrogliosis indicated by increased expression of *GFAP* [46]. Inflammation and immunity seem to be the only major functional group with sex differences. However, also in males, inflammation and immune responses are activated, thus confirming the results reported by Brink et al. [8].

This study may be limited by potential technical inaccuracies including differences between platforms that may not fully be equalized by cross-platform-normalization and gene expression changes in the post-mortem interval. Furthermore, causality tested by the Granger test refers to the ability of prior values of one time series to predict values of another time series that may not be necessarily causative. For the explanatory power of the time series one has to take into account the construction from multiple individuals.

In this sex -specific meta-analysis of PFC biopsy-derived transcriptomes, we uncovered gene sets positively and negatively correlated with age which eventually could be condensed to similar functionality in both sexes. Synaptic transmission was found to be most significantly down-regulated with age while the expression of the reactive astrocyte marker *GFAP* was the most significantly up–regulated gene with age. However, many more players are involved in the complex mechanisms of brain aging. We identified age-associated downregulated expression of *CAMK4* - potentially contributing to up-regulation of *GFAP* - and Calcium signaling, hormones, insulin secretion, cAMP, long-term potentiation, neurogenesis and dendritic spines declining with age. On the other hand, inflammation, oxidative stress and neuronal injury increased with age. In summary, we found that during aging synaptic transmission declines due to a complex interplay of increasing factors such as inflammation, oxidative stress, nitric oxide and decreasing factors such as calcium signaling, cAMP, dendritic spines, long-term potentiation, hormones and CCL2. These findings are summarized in the scheme presented in Fig. 7b.

The dataset provided here should be useful for experimentalist to test and derive novel hypothesis on brain aging using iPSC-based tools.

## Supplementary information

**Additional file 1: Supplementary Figure 1:** Characteristics of PCA. (A) Correlation plot of variances of genes influencing PC1 the most (B) Correlation plot of variances of genes influencing PC2 the most. (C) Scree plot of variances against the most important principal components (D) Variances of genes influencing PC1 the most. (E) Variances of genes influencing PC2 the most.

**Additional file 2: Supplementary Table 1.** Prefrontal cortex transcriptome datasets and their GEO accession numbers employed for the meta-analysis.

**Additional file 3: Supplementary Table 2.** Results of statistical tests comparing female versus male PFC biopsy-derived gene expression data. *P*-values and q-values are based on the R packages limma and qvalue, ratios are calculated by dividing mean female by mean male expression.

**Additional file 4: Supplementary Table 3.** Subsets of the venn diagrams comparing male and female differentially up- and down-regulated genes in young, middle-aged and old (sheets as in Fig. 2). (A) Genes down-regulated in F30_65 vs. F30 were compared with genes down-regulated in M30_65 vs. M30. (B) Genes up-regulated in F30_65 vs. F30 were compared with genes up-regulated in M30_65 vs. M30. (C) Genes down-regulated in F65 vs. F30_65 were compared with genes down-regulated in M65 vs. M30_65. (D) Genes up-regulated in F65 vs. F30_65 were compared with genes up-regulated in M65 vs. M30_65. (E) Genes down-regulated in F65 vs. F30 were compared with genes down-regulated in M65 vs. M30. (F) Genes up-regulated in F65 vs. F30 were compared with genes up-regulated in M65 vs. M30.

**Additional file 5: Supplementary Table 4.** Correlation of gene expression with age. (A) Pearson correlation coefficients of gene expression with age and corresponding *p*-value, q-values and Bonferroni corrected *p*-values. (B) 48 genes with highest differences in age correlation between female and male. Genes with higher age correlation in male than in female are marked red in column *cor_M-F*, with lower age correlation in green.

**Additional file 6: Supplementary Table 5.** Over-represented GO terms in genes anti-correlated and correlated with age. (A) anti-correlated in female, (B) anti-correlated in male, (C) correlated in female, (D) correlated in male. Genes for GO analysis were filtered with Bonferroni-corrected *p* < 0.05 and *r* < − 0.1 for anti-correlated genes or *r* > 0.1 for correlated genes.

**Additional file 7: Supplementary Table 6.** Over-represented KEGG pathways in genes anti-correlated and correlated with age. (A) anti-correlated in female, (B) anti-correlated in male, (C) correlated in female, (D) correlated in male. Genes for GO analysis were filtered with Bonferroni-corrected *p* < 0.05 and *r* < − 0.1 for anti-correlated genes or *r* > 0.1 for correlated genes.

**Additional file 8: Supplementary Table 7.** Trancription factors in the GFAP 2 k base upstream region found with a p-Match search of the public Transfac database filtering with core-d-score < 0.9 and matrix-d-score < 0.9

## Data Availability

In this meta-analysis publicly available datasets from NCBI GEO were used. Generated data is submitted as supplementary material.

## References

[1] Akintola AA, van Heemst D (2015). Insulin, aging, and the brain: mechanisms and implications. Front Endocrinol.

[2] Altman J, Das GD (1965). Autoradiographic and histological evidence of postnatal hippocampal neurogenesis in rats. J Comp Neurol.

[3] Arnold SE, Arvanitakis Z, Macauley-Rambach SL, Koenig AM, Wang H-Y, Ahima RS, Craft S, Gandy S, Buettner C, Stoeckel LE, Holtzman DM, Nathan DM (2018). Brain insulin resistance in type 2 diabetes and Alzheimer disease: concepts and conundrums. Nat Rev Neurol.

[4] Barnes MR, Huxley-Jones J, Maycox PR, Lennon M, Thornber A, Kelly F, Bates S, Taylor A, Reid J, Jones N, Schroeder J, Scorer CA, Davies C, Hagan JJ, Kew JNC, Angelinetta C, Akbar T, Hirsch S, Mortimer AM, Barnes TRE, de Belleroche J (2011). Transcription and pathway analysis of the superior temporal cortex and anterior prefrontal cortex in schizophrenia. J Neurosci Res.

[5] Bito H, Deisseroth K, Tsien RW (1996). CREB phosphorylation and dephosphorylation: a Ca(2+)- and stimulus duration-dependent switch for hippocampal gene expression. Cell.

[6] Bourne J, Harris KM (2007). Do thin spines learn to be mushroom spines that remember?. Curr Opin Neurobiol.

[7] Brink TC, Demetrius L, Lehrach H, Adjaye J (2009). Age-related transcriptional changes in gene expression in different organs of mice support the metabolic stability theory of aging. Biogerontology.

[8] Brink TC, Regenbrecht C, Demetrius L, Lehrach H, Adjaye J (2009). Activation of the immune response is a key feature of aging in mice. Biogerontology.

[9] Chatr-Aryamontri A, Oughtred R, Boucher L, Rust J, Chang C, Kolas NK, O’Donnell L, Oster S, Theesfeld C, Sellam A, Stark C, Breitkreutz B-J, Dolinski K, Tyers M (2017). The BioGRID interaction database: 2017 update. Nucleic Acids Res.

[10] Chen C-Y, Logan RW, Ma T, Lewis DA, Tseng GC, Sibille E, McClung CA (2016). Effects of aging on circadian patterns of gene expression in the human prefrontal cortex. Proc Natl Acad Sci U S A.

[11] Cheng H, Xuan H, Green CD, Han Y, Sun N, Shen H, McDermott J, Bennett DA, Lan F, Han J-DJ (2018). Repression of human and mouse brain inflammaging transcriptome by broad gene-body histone hyperacetylation. Proc Natl Acad Sci U S A.

[12] Csardi G, Nepusz T (2006). The igraph software package for complex network research. InterJ Complex Syst.

[13] de la Monte SM, Wands JR (2008). Alzheimer’s disease is type 3 diabetes--evidence reviewed. J Diabetes Sci Technol.

[14] El Khoury J, Toft M, Hickman SE, Means TK, Terada K, Geula C, Luster AD (2007). Ccr2 deficiency impairs microglial accumulation and accelerates progression of Alzheimer-like disease. Nat Med.

[15] Eugenin EA, D’Aversa TG, Lopez L, Calderon TM, Berman JW (2003). MCP-1 (CCL2) protects human neurons and astrocytes from NMDA or HIV-tat-induced apoptosis. J Neurochem.

[16] Falcon S, Gentleman R (2007). Using GOstats to test gene lists for GO term association. Bioinformatics.

[17] Frölich L, Blum-Degen D, Bernstein HG, Engelsberger S, Humrich J, Laufer S, Muschner D, Thalheimer A, Türk A, Hoyer S, Zöchling R, Boissl KW, Jellinger K, Riederer P (1998). Brain insulin and insulin receptors in aging and sporadic Alzheimer’s disease. J Neural Transm (Vienna).

[18] Furman B, Ong WK, Pyne NJ (2010). Cyclic AMP signaling in pancreatic islets. Adv Exp Med Biol.

[19] Galili T (2015). Dendextend: an R package for visualizing, adjusting and comparing trees of hierarchical clustering. Bioinformatics.

[20] Garelick T, Swann J (2014). Testosterone regulates the density of dendritic spines in the male preoptic area. Horm Behav.

[21] Gentleman RC, Carey VJ, Bates DM, Bolstad B, Dettling M, Dudoit S, Ellis B, Gautier L, Ge Y, Gentry J, Hornik K, Hothorn T, Huber W, Iacus S, Irizarry R, Leisch F, Li C, Maechler M, Rossini AJ, Sawitzki G, Smith C, Smyth G, Tierney L, Yang JYH, Zhang J (2004). Bioconductor: open software development for computational biology and bioinformatics. Genome Biol.

[22] Granger CWJ (1969). Investigating causal relations by econometric models and cross-spectral methods. Econometrica.

[23] Gray SM, Meijer RI, Barrett EJ (2014). Insulin regulates brain function, but how does it get there?. Diabetes.

[24] Hagenauer MH, Schulmann A, Li JZ, Vawter MP, Walsh DM, Thompson RC, Turner CA, Bunney WE, Myers RM, Barchas JD, Schatzberg AF, Watson SJ, Akil H (2018). Inference of cell type content from human brain transcriptomic datasets illuminates the effects of age, manner of death, dissection, and psychiatric diagnosis. PLoS One.

[25] Harris KM, Kater SB (1994). Dendritic spines: cellular specializations imparting both stability and flexibility to synaptic function. Annu Rev Neurosci.

[26] Hekimi S, Lapointe J, Wen Y (2011). Taking a “good” look at free radicals in the aging process. Trends Cell Biol.

[27] Heppner FL, Ransohoff RM, Becher B (2015). Immune attack: the role of inflammation in Alzheimer disease. Nat Rev Neurosci.

[28] Hyndman RJ, Khandakar Y (2008). Automatic time series forecasting: the forecast package for R. J Stat Softw.

[29] Kanehisa M, Furumichi M, Tanabe M, Sato Y, Morishima K (2017). KEGG: new perspectives on genomes, pathways, diseases and drugs. Nucleic Acids Res.

[30] Kassambara A (2017). Practical guide to cluster analysis in R: unsupervised machine learning, edition 1.

[31] Knott AB, Bossy-Wetzel E (2010). Impact of nitric oxide on metabolism in health and age-related disease. Diabetes Obes Metab.

[32] Kohman RA, Rhodes JS (2013). Neurogenesis, inflammation and behavior. Brain Behav Immun.

[33] Kumar A, Bodhinathan K, Foster TC (2009). Susceptibility to calcium dysregulation during brain aging. Front Aging Neurosci.

[34] Lanz TA, Joshi JJ, Reinhart V, Johnson K, Grantham LE, Volfson D (2015). STEP levels are unchanged in pre-frontal cortex and associative striatum in post-mortem human brain samples from subjects with schizophrenia, bipolar disorder and major depressive disorder. PLoS One.

[35] López-Otín C, Blasco MA, Partridge L, Serrano M, Kroemer G (2013). The hallmarks of aging. Cell.

[36] Lu T, Aron L, Zullo J, Pan Y, Kim H, Chen Y, Yang T-H, Kim H-M, Drake D, Liu XS, Bennett DA, Colaiácovo MP, Yankner BA (2014). REST and stress resistance in ageing and Alzheimer’s disease. Nature.

[37] Manganas LN, Zhang X, Li Y, Hazel RD, Smith SD, Wagshul ME, Henn F, Benveniste H, Djuric PM, Enikolopov G, Maletic-Savatic M (2007). Magnetic resonance spectroscopy identifies neural progenitor cells in the live human brain. Science.

[38] Middeldorp J, Hol EM (2011). GFAP in health and disease. Prog Neurobiol.

[39] Morrison JH, Baxter MG (2012). The ageing cortical synapse: hallmarks and implications for cognitive decline. Nat Rev Neurosci.

[40] Mostany R, Anstey JE, Crump KL, Maco B, Knott G, Portera-Cailliau C (2013). Altered synaptic dynamics during normal brain aging. J Neurosci.

[41] Narayan S, Tang B, Head SR, Gilmartin TJ, Sutcliffe JG, Dean B, Thomas EA (2008). Molecular profiles of schizophrenia in the CNS at different stages of illness. Brain Res.

[42] Park S-J, Ahmad F, Philp A, Baar K, Williams T, Luo H, Ke H, Rehmann H, Taussig R, Brown AL, Kim MK, Beaven MA, Burgin AB, Manganiello V, Chung JH (2012). Resveratrol ameliorates aging-related metabolic phenotypes by inhibiting cAMP phosphodiesterases. Cell.

[43] Pugazhenthi S, Wang M, Pham S, Sze C-I, Eckman CB (2011). Downregulation of CREB expression in Alzheimer’s brain and in Aβ-treated rat hippocampal neurons. Mol Neurodegener.

[44] Schmidt A, Wolde M, Thiele C, Fest W, Kratzin H, Podtelejnikov AV, Witke W, Huttner WB, Söling HD (1999). Endophilin I mediates synaptic vesicle formation by transfer of arachidonate to lysophosphatidic acid. Nature.

[45] Smith CC, Vedder LC, McMahon LL (2009). Estradiol and the relationship between dendritic spines, NR2B containing NMDA receptors, and the magnitude of long-term potentiation at hippocampal CA3-CA1 synapses. Psychoneuroendocrinology.

[46] Sofroniew MV, Vinters HV (2010). Astrocytes: biology and pathology. Acta Neuropathol.

[47] Sticozzi C, Belmonte G, Meini A, Carbotti P, Grasso G, Palmi M (2013). IL-1β induces GFAP expression in vitro and in vivo and protects neurons from traumatic injury-associated apoptosis in rat brain striatum via NFκB/Ca^2+^-calmodulin/ERK mitogen-activated protein kinase signaling pathway. Neuroscience.

[48] Taminau J, Meganck S, Lazar C, Steenhoff D, Coletta A, Molter C, Duque R, de Schaetzen V, Weiss Solís DY, Bersini H, Nowé A (2012). Unlocking the potential of publicly available microarray data using inSilicoDb and inSilicoMerging R/bioconductor packages. BMC Bioinformatics.

[49] Wang Z, Zhang L, Liang Y, Zhang C, Xu Z, Zhang L, Fuji R, Mu W, Li L, Jiang J, Ju Y, Wang Z (2015). Cyclic AMP mimics the anti-ageing effects of calorie restriction by up-regulating Sirtuin. Sci Rep.

[50] Wei T, Simko V (2017). R package “corrplot”: visualization of a correlation matrix.

[51] Wickham H (2009). Ggplot2: elegant graphics for data analysis.

[52] Woolley CS, McEwen BS (1994). Estradiol regulates hippocampal dendritic spine density via an N-methyl-D-aspartate receptor-dependent mechanism. J Neurosci.

[53] Wruck W, Schröter F, Adjaye J (2016). Meta-analysis of transcriptome data related to hippocampus biopsies and iPSC-derived neuronal cells from alzheimer’s disease patients reveals an association with FOXA1 and FOXA2 gene regulatory networks. J Alzheimers Dis.

[54] Wu GY, Deisseroth K, Tsien RW (2001). Activity-dependent CREB phosphorylation: convergence of a fast, sensitive calmodulin kinase pathway and a slow, less sensitive mitogen-activated protein kinase pathway. Proc Natl Acad Sci U S A.

[55] Wu L-S, Cheng W-C, Chen C-Y, Wu M-C, Wang Y-C, Tseng Y-H, Chuang T-J, Shen C-KJ (2019). Transcriptomopathies of pre- and post-symptomatic frontotemporal dementia-like mice with TDP-43 depletion in forebrain neurons. Acta Neuropathol Commun.

[56] Zeileis A, Hothorn T (2002). Diagnostic checking in regression relationships. R News.

