# Supplementary material for: Meta-analysis of human prefrontal cortex reveals activation of GFAP and decline of synaptic transmission in the aging brain
Source: Acta Neuropathol Commun. 2020 Mar 5;8:26. doi: 10.1186/s40478-020-00907-8 (PMC7059712; doi:10.1186/s40478-020-00907-8)
Supplement: Supplementary file 1 — Additional file 1: Supplementary Figure 1: Characteristics of PCA. (A) Correlation plot of variances of genes influencing PC1 the most (B) Correlation plot of variances of genes influencing PC2 the most. (C) Scree plot of variances against the most important principal components (D) Variances of genes influencing PC1 the most. (E) Variances of genes influencing PC2 the most. [file 40478_2020_907_MOESM1_ESM.pdf]

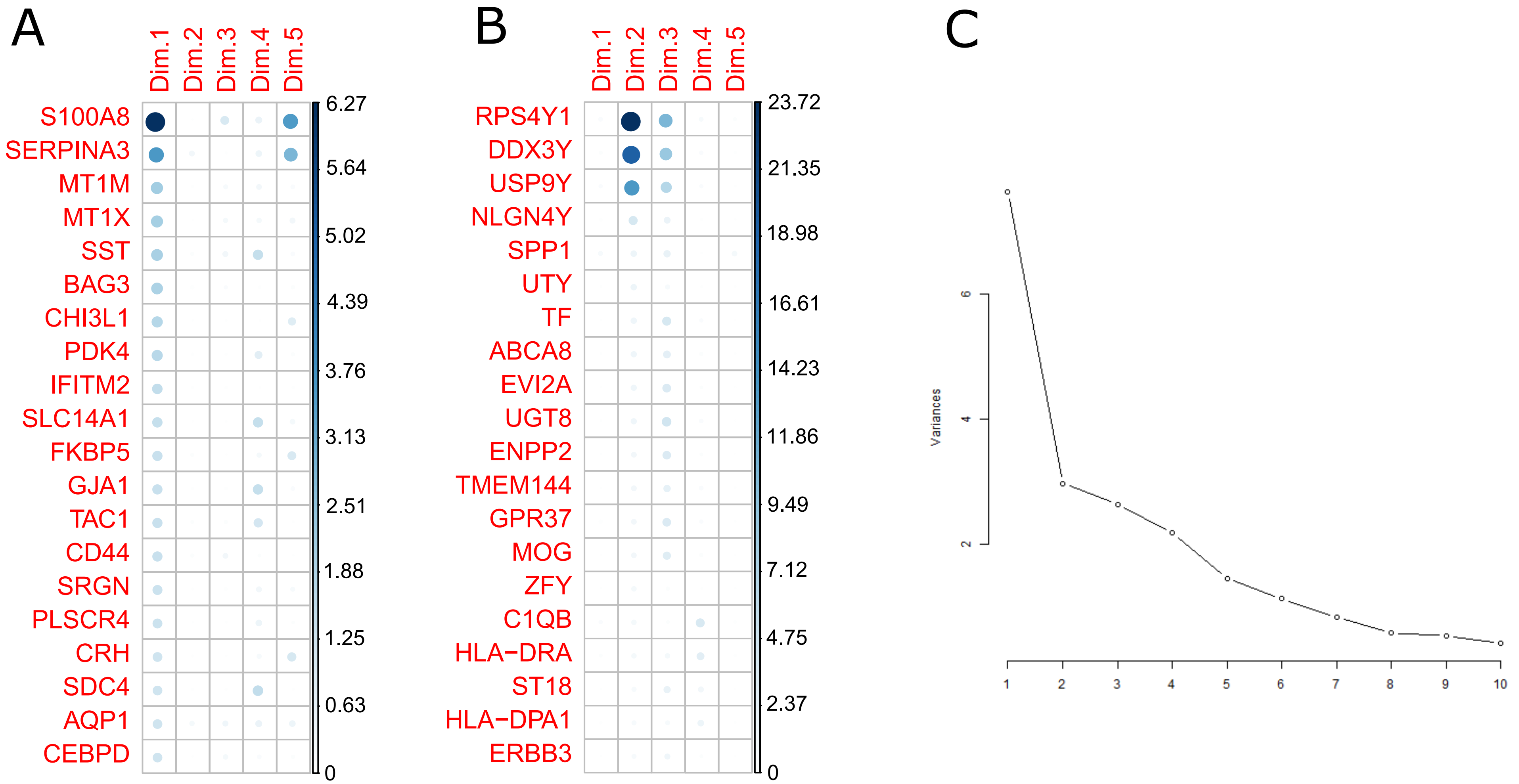

D

gene_symbol	Dim.1	Dim.2	Dim.3	Dim.4	Dim.5
S100A8	6.2690	0.0220	1.0233	0.5463	3.5251
SERPINA3	3.5972	0.3551	0.0205	0.4842	2.8734
MT1M	2.1805	0.0360	0.2084	0.3249	0.1840
MT1X	2.1239	0.0079	0.2558	0.2743	0.3684
SST	2.0194	0.1182	0.3736	1.4717	0.1761
BAG3	1.9941	0.0000	0.1259	0.0470	0.1252
CHI3L1	1.7840	0.0106	0.0099	0.0044	0.8403
PDK4	1.7546	0.0346	0.0248	0.7851	0.1105
IFITM2	1.5297	0.0291	0.0343	0.0047	0.0332
SLC14A1	1.4847	0.0036	0.0096	1.4546	0.3093
FKBP5	1.4310	0.1070	0.0024	0.2531	1.0222
GJA1	1.4301	0.0340	0.0497	1.4747	0.1643
TAC1	1.4276	0.0960	0.1137	1.1422	0.0118
CD44	1.4276	0.1211	0.3327	0.0785	0.0126
SRGN	1.3523	0.0214	0.0610	0.3664	0.2184
PLSCR4	1.3225	0.0042	0.0111	0.4904	0.0632
CRH	1.2784	0.0168	0.0066	0.2431	1.1179
SDC4	1.2660	0.0430	0.0241	1.5592	0.0153
AQP1	1.2641	0.2724	0.3780	0.5485	0.4945
CEBPD	1.2630	0.0149	0.2802	0.1829	0.1136

E

gene_symbol	Dim.1	Dim.2	Dim.3	Dim.4	Dim.5
RPS4Y1	0.6976	23.7233	10.7864	0.7239	0.4154
DDX3Y	0.4322	19.3237	8.9254	0.6050	0.1645
USP9Y	0.5062	13.5471	6.6884	0.4785	0.0751
NLGN4Y	0.2212	4.0794	1.9833	0.0922	0.0172
SPP1	0.8371	1.7525	2.3601	0.0407	1.1837
UTY	0.0546	1.6769	0.8757	0.0783	0.0375
TF	0.0145	1.6525	4.0883	0.4877	0.0013
ABCA8	0.0568	1.5887	2.8474	0.4131	0.0365
EVI2A	0.0023	1.5511	3.7543	0.2945	0.0099
UGT8	0.0228	1.5361	4.6141	0.6467	0.0076
ENPP2	0.0235	1.2637	3.6088	0.3208	0.0027
TMEM144	0.0274	1.2501	2.4426	0.7034	0.0451
GPR37	0.0009	1.2361	3.7036	0.4865	0.0449
MOG	0.0026	1.2077	3.1716	0.4647	0.0074
ZFY	0.0211	1.0903	0.4510	0.0562	0.0202
C1QB	0.5013	1.0797	0.3334	4.0094	0.2238
HLA-DRA	0.3196	1.0634	1.0555	2.8860	0.1734
ST18	0.0264	0.9252	2.2961	1.4034	0.0043
HLA-DPA1	0.1856	0.8732	0.8058	1.8726	0.0599
ERBB3	0.0090	0.8264	1.4320	0.3724	0.0013
