# Supplementary material for: Meta-analysis of human prefrontal cortex reveals activation of GFAP and decline of synaptic transmission in the aging brain
Source: Acta Neuropathol Commun. 2020 Mar 5;8:26. doi: 10.1186/s40478-020-00907-8 (PMC7059712; doi:10.1186/s40478-020-00907-8)
Supplement: Supplementary file 2 — Additional file 2: Supplementary Table 1. Prefrontal cortex transcriptome datasets and their GEO accession numbers employed for the meta-analysis. [file 40478_2020_907_MOESM2_ESM.docx]

| **dataset** | **contents** | **brain region** | **platform** | **PubMedId** | **comment** |
| --- | --- | --- | --- | --- | --- |
| GSE21138 | shizophrenia and control | prefrontal cortex | Affymetrix Human Genome U133 Plus 2.0 | [18778695](https://www.ncbi.nlm.nih.gov/pubmed/18778695) |  |
| GSE21935 | shizophrenia and control | anterior prefrontal cortex (BA10) and superior temporal cortex (BA22) | Affymetrix Human Genome U133 Plus 2.0 | [21538462](https://www.ncbi.nlm.nih.gov/pubmed/21538462) |  |
| GSE53890 | control | frontal cortex | Affymetrix Human Genome U133 Plus 2.0 | [24670762](https://www.ncbi.nlm.nih.gov/pubmed/24670762) | Comparison of the gene expression profiles of adult human brain samples from frontal cortical regions, including samples from young, middle aged, normal aged. |
| GSE53987 | schizophrenia, bipolar disorder, major depressive disorder and matched controls | Pre-frontal cortex, striatum and hippocampus | Affymetrix Human Genome U133 Plus 2.0 | [25786133](https://www.ncbi.nlm.nih.gov/pubmed/25786133) | Post-mortem brain tissue was collected from control subjects and well-matched subjects with schizophrenia, BPD, and MDD (n=19 from each group). RNA was isolated from hippocampus, Brodmann Area 46, and associative striatum and hybridized to U133_Plus2 Affymetrix chips. |
| GSE71620 | control | human prefrontal cortex (Brodmann’s areas (BA) 11 and 47) | Affymetrix Human Gene 1.1 ST | [26699485](https://www.ncbi.nlm.nih.gov/pubmed/26699485) |  |
| GSE92538 | control, Schizophrenia, MajorDepressiveDisorder,BipolarDisorder | dorsolateral prefrontal cortex (DLPFC) | Affymetrix Human Genome U133 Plus 2.0 | [30016334](https://www.ncbi.nlm.nih.gov/pubmed/30016334) |  |
| GSE106669 | control | prefrontal cortex | Illumina HiSeq 2000 (Homo sapiens) | [29967166](https://www.ncbi.nlm.nih.gov/pubmed/29967166) | For RNA extraction, human brain samples with similar age were pooled together |

Supplementary Table 1
